# Evaluating the Validity of an Automated Device for Asthma Monitoring for Adolescents: Correlational Design

**DOI:** 10.2196/jmir.4975

**Published:** 2015-10-16

**Authors:** Hyekyun Rhee, Michael J Belyea, Mark Sterling, Mark F Bocko

**Affiliations:** ^1^School of NursingUniversity of RochesterRochester, NYUnited States; ^2^College of Nursing and Health InnovationArizona State UniversityPhoenix, AZUnited States; ^3^Department of Computer ScienceSchool of Science and TechnologyNazarbayev UniversityAstanaKazakhstan; ^4^Department of Electrical and Computer EngineeringUniversity of RochesterRochester, NYUnited States

**Keywords:** asthma, adolescent, ambulatory monitoring, device, cough, validity

## Abstract

**Background:**

Symptom monitoring is a cornerstone of asthma self-management. Conventional methods of symptom monitoring have fallen short in producing objective data and eliciting patients’ consistent adherence, particularly in teen patients. We have recently developed an Automated Device for Asthma Monitoring (ADAM) using a consumer mobile device as a platform to facilitate continuous and objective symptom monitoring in adolescents in vivo.

**Objective:**

The objectives of the study were to evaluate the validity of the device using spirometer data, fractional exhaled nitric oxide (FeNO), existing measures of asthma symptoms/control and health care utilization data, and to examine the sensitivity and specificity of the device in discriminating asthma cases from nonasthma cases.

**Methods:**

A total of 84 teens (42 teens with a current asthma diagnosis; 42 without asthma) aged between 13 and 17 years participated in the study. All participants used ADAM for 7 consecutive days during which participants with asthma completed an asthma diary two times a day. ADAM recorded the frequency of coughing for 24 hours throughout the 7-day trial. Pearson correlation and multiple regression were used to examine the relationships between ADAM data and asthma control, quality of life, and health care utilization at the time of the 7-day trial and 3 months later. A receiver operating characteristic (ROC) curve analysis was conducted to examine sensitivity and specificity based on the area under the curve (AUC) as an indicator of the device’s capacity to discriminate between asthma versus nonasthma cases.

**Results:**

ADAM data (cough counts) were negatively associated with forced expiratory volume in first second of expiration (FEV_1_) (*r*=–.26, *P*=.05), forced vital capacity (FVC) (*r*=–.31, *P*=.02), and overall asthma control (*r*=–.41, *P*=.009) and positively associated with daily activity limitation (*r*=.46, *P*=.01), nighttime (*r*=.40, *P*=.02) and daytime symptoms (*r*=.38, *P*=.02), and health care utilization (*r*=.61, *P*<.001). Device data were also a significant predictor of asthma control (β=–.48, *P*=.003), quality of life (β=–.55, *P*=.001), and health care utilization (β=.74, *P*=.004) after 3 months. The ROC curve analysis for the presence of asthma diagnosis had an AUC of 0.71 (95% CI 0.58-0.84), which was significantly different from chance (χ^2^_1_=9.7, *P*=.002), indicating the device’s discriminating capacity. The optimal cutoff value of the device was 0.56 with a sensitivity of 51.3% and a specificity of 72.7%.

**Conclusions:**

This study demonstrates validity of ADAM as a symptom-monitoring device in teens with asthma. ADAM data reflect the current status of asthma control and predict asthma morbidity and quality of life for the near future. A monitoring device such as ADAM can increase patients’ awareness of the patterns of cough for early detection of worsening asthma and has the potential for preventing serious and costly future consequences of asthma.

## Introduction

Achieving acceptable asthma control in adolescents remains elusive despite the availability of efficacious treatment options. In 2010, nearly 11% of adolescents (2.7 million) aged 12-17 years in the United States reported a current diagnosis of asthma [1] and adolescents suffer greater asthma-related morbidity than other age groups [2]. Adverse asthma outcomes in this age group are largely attributable to poor self-management [3-5]. Establishing daily routines of symptom monitoring is recognized as the initial step to successful asthma self-management [6,7] leading to better asthma outcomes [8].

National Heart, Lung, and Blood Institute (NHLBI) Expert Panel Review 3 (EPR 3) [9] recommends that all patients with asthma learn to recognize symptom patterns related to inadequate asthma control. Currently, there are 2 basic types of home-based symptom-monitoring methods that patients can use to monitor symptom patterns: symptom-based and peak flow monitoring. However, many studies have consistently raised a concern about young people’s poor perception in recognizing asthma symptoms [10-17]. Adolescents, in particular, tend to be overly optimistic in rating their asthma control despite the presence of symptoms and activity limitations [18-20]. As a consequence, sole reliance on patients’ perception in symptom monitoring can be misleading.

Peak flow monitoring has been recommended to enhance the objectivity of symptom monitoring, particularly for those who suffer a high level of asthma severity because of their impaired symptom perception [21-23]. However, confirming the reliability of peak expiratory flow rate values has been an ongoing challenge [24-26]. Moreover, its clinical usefulness in children and adolescents is hampered by users’ poor adherence and inadequate techniques, and the effort-dependent nature of the method. Concerns have been raised about questionable long-term sustainability of peak flow monitoring and inaccurate and/or fabricated readings [27-32].

Given the limitations of the existing symptom-monitoring methods, alternative strategies have been called for to mitigate the previously mentioned issues and enhance objectivity and sustainability for continuous symptom monitoring in children and adolescents [8,29,32,33]. Recently, we developed an Automated Device for Asthma Monitoring (ADAM) to increase the objectivity of symptom monitoring and to facilitate adolescents’ adherence to continuous symptom monitoring in vivo. The device employed audio analysis technology to recognize symptoms, particularly coughs. The device uses a mobile system, iPod, as a platform. The methodological and technical details involving the development of the device and user acceptability are reported elsewhere [34,35]. This paper reports findings on the validity of the device as a monitoring tool. Specifically, this study examined (1) concurrent validity by correlating the data of the device with other measurements informing asthma control, including pulmonary function, fractional exhaled nitric oxide (FeNO), symptom-based monitoring (eg, daily asthma diary), asthma control questions, quality of life, and health care utilization; (2) predictive validity of the device by examining the extent to which the results of the device predict asthma control, quality of life, and health care utilization after 3 months; and (3) sensitivity and specificity of the device in discriminating between an asthma group and a nonasthma group.

## Methods

### Study Sample and Setting

Participant eligibility criteria for the asthma group were (1) age 13-17 years, (2) physician-diagnosed asthma for at least 1 year, and (3) ability to understand spoken and written English. The nonasthma group were age-matched adolescents with no current/past history of asthma and free of ongoing respiratory conditions. For both groups, we excluded those with other diagnoses producing respiratory symptoms (eg, upper respiratory infection, cardiac disease, cystic fibrosis) or significant cognitive impairment that could interfere with following the study protocol. Potential participants were recruited from the pediatric emergency department (ED) and outpatient clinics (primary practice and pediatric pulmonary practice) in a major university medical center located in the Northeastern United States. Of a total of 84 participants, most (73%, 61/84) were recruited from the ED and the remaining were from the study flyers (23%, 19/84) and clinician referrals from outpatient clinics (4%, 4/84). Unverifiable asthma diagnosis by medical records was the most common cause of ineligibility for the asthma group (n=52) followed by comorbidity with other respiratory diagnosis (n=14). For the nonasthma group, having an asthma diagnosis in the past (n=22) and presenting respiratory symptoms (n=7) were common reasons for ineligibility.

### Study Measures: Both Groups

#### Automated Device for Asthma Monitoring

The ADAM device uses an iPod as a platform and was designed to continuously process audio data in real time to detect coughs. The device detected the number of cough events in 6-second intervals. It also provided a display of cough count data in a chart form on the device for users. Detailed descriptions of the device have been reported elsewhere [34,35]. ADAM was used by all participants for at least 7 consecutive days.

#### Fractional Exhaled Nitric Oxide

FeNO is a noninvasive method of assessing asthmatic inflammation [36]. Increasing FeNO levels have been found to be predictive of deteriorating asthma [37] and correlates more closely with symptoms than does forced expiratory volume in the first second (FEV_1_) [38,39]. FeNO was measured before and after the 7-day trial in accordance with the American Thoracic Society (ATS) recommendations [40] using NIOX MINO (Aerocrine AB, Stockholm, Sweden).

#### Pulmonary Function Test

To assess the degree of airway obstruction, the volume of air expired during the first second of a forced vital capacity maneuver (FEV_1_) and forced vital capacity (FVC) was measured using a KoKo spirometer (Pulmonary Data Service; Louisville, CO, USA) connected to a personal computer. Trained research staff performed spirometry for each participant two times, before and after the 7-day trial in accordance with the ATS standards [41].

#### Participant Demographic Form

Basic demographic information was collected, including gender, age, race, annual family income, years with asthma diagnosis (for the asthma group), types of health conditions that led to a clinic or ED visit (for the comparison group), and current medications (if applicable).

### Study Measures: Asthma Group Only

#### Asthma Control Questions

The Asthma Control Questionnaire (ACQ) was developed based on the 2007 NHLBI National Guidelines’ asthma control classification criteria involving 4 areas of asthma impairment, including the frequency of daily activity limitations, asthma symptoms, nighttime symptoms, and use of short-acting beta agonists (SABA) in the past 4 weeks. The 4 questions were measured on a 5-point scale and higher total scores indicated better asthma control. The ACQ was administered at pretrial and at 6-month follow-up. Cronbach alpha of the scale was .79 in this study.

#### Pediatric Asthma Quality of Life Questionnaire

The 23-item Pediatric Asthma Quality of Life Questionnaire (PAQLQ) measures 3 subdomains pertaining to asthma-related quality of life in children with asthma aged 7-17 years, including symptoms (10 items), emotional function (8 items), and activity limitation (5 items) [42]. Each item is measured on a 7-point scale (1=maximum impairment, 7=no impairment). Higher total scores indicate better levels of functioning. In this study, high internal consistency (Cronbach alpha) was found in all 3 subscales: .94, .95, and .88 for symptoms, emotional function, and activity limitation subdomains, respectively.

#### Health Care Utilization Form

The Health Care Utilization Form captured any asthma-related events including ED visits, hospitalization, office visits, and missed school days. This form assessed the frequency of events that occurred over the past 3 months (pretrial, 3-month follow-up) and the past 7 days for post-trial.

#### Visual Analog Scale

The Visual Analog Scale (VAS) is a line 100 mm long with 3 anchors dividing 3 zones (red, yellow, and green). For each symptom, there is a green zone (80-100 mm) labeled “no symptoms,” a yellow zone (79-50 mm) labeled “mild symptoms,” and a red zone (<50 mm) labeled “very bad symptoms” [16,29]. Teens marked any point on the line according to their perception of asthma symptoms two times a day in the morning and evening during the 7-day trial. The distance between the 0-mm mark and the placement of the “X” was measured to provide a numeric interpretation of their symptom perception.

#### Asthma Control Diary

The Asthma Control Diary consisted of 6 items, each with scores ranging from 0 (no symptoms) to 6 (continual symptoms) [43]. The device automatically sent diary reminders two times a day during the 7-day trial and allowed teens to conveniently complete the diary electronically using the touchscreen. The device automatically triggered diary reminders and made diary questions available only within the designated time window for am (6 am-noon) or pm (6 pm-midnight) to minimize recollection errors or the risk of data fabrication later. Morning questions pertained to nocturnal waking and morning symptoms, and evening questions assessed the degree of activity limitation, daytime symptoms (shortness of breath and wheeze), and SABA use in previous 24 hours. The mean score for each diary question was computed with higher scores indicating a greater degree of symptoms.

### Study Procedure

At enrollment, spirometry and FeNO tests were conducted for all participants followed by the measurement of asthma control and health care utilization for the asthma group. All participants used the device continuously for the next 7 days during which the asthma group completed daily the electronic asthma diary and the VAS in the morning and at bedtime. On completion of the 7-day trial, spirometry and FeNO tests were repeated for all participants. In addition, the asthma group completed a quality of life questionnaire and reported any health care utilization that occurred in the past week. Follow-up data on asthma control, health care utilization, and quality of life were collected at 3 months after the trial only from the asthma group. Only 2 of 42 participants in the asthma group were lost to the 3-month follow-up. Of the 40 follow-up cases, 30 were completed by mail and 10 were in-person with research staff. This study protocol was approved by the Institutional Review Board, the Research Subjects Review Board, located in the University of Rochester Medical Center, Rochester, NY, and informed consent and assent were obtained from parents and teens, respectively. The participants received a monetary incentive (US $130 for the asthma group; US $100 for the nonasthma group) for their participation.

### Data Analysis

All analyses were performed using SAS v9.3 (SAS Institute, Inc, Cary, NC, USA). Descriptive statistics were used to examine demographic and clinical characteristics. To assess concurrent validity, Pearson correlations were calculated between the device data from the asthma group with spirometer data (FEV_1_ and FVC), FeNO, and other measures of asthma conditions, including asthma control questions, daily symptom diaries, VAS, quality of life, and health care utilization. To assess predictive validity of the device, regression was used to examine the relationships between the device data and asthma control, health care utilization, and quality of life collected at 3 months after the 7-day trial. Age, gender, and race were adjusted for in the regression analyses. Sensitivity and specificity were evaluated by assessing the device’s capability to classify 2 distinctive groups. A receiver operating characteristic (ROC) curve analysis was conducted to calculate the area under the curve (AUC) as an indicator of the device’s capacity to discriminate between asthma and nonasthma participants. An AUC of 1 indicates perfect classification and an AUC of 0.5 indicates that the ability to correctly classify is no better than chance. Based on the ROC, an optimum cutoff value was chosen to jointly maximize the sensitivity and specificity of the device. Given the few studies looking at devices for symptom monitoring in adolescents, any noticeable statistical trend is valuable; therefore, the significance level used in the analysis was less than .10. These findings are useful for an initial understanding of the validity of ADAM and for providing direction for further studies. Although it is understood that these findings will require further investigation, the risk of rejecting important research hypotheses was judged more important than the risk of type I error.

## Results

### Sample Characteristics

Details of participant flow from screening to the 3-month follow-up and sociodemographic characteristics of each group in the sample are reported elsewhere [34]. A total of 84 adolescents aged between 13 and 17 years (mean 15 years, SD 1.4) participated in the study. Of those, 61% (51/84) were females and 44% (37/84) were nonwhite adolescents. No significant differences were found between the asthma and nonasthma groups in age and gender. The asthma group included significantly more nonwhite adolescents, predominantly African American. At enrollment, the asthma group had slightly lower FEV_1_ (mean 88.3%, SD 16.3 vs mean 90.9%, SD 13.8) and elevated FeNO (mean 28.6 ppb, SD 38.6 vs mean 25.6 ppb, SD 24.8) than the nonasthma group, yet these differences were not statistically significant. Within the asthma group, 19 of 42 (45%) reported active asthma symptoms at enrollment. Mean years since asthma diagnosis was 10.4 (SD 4.9) years. SABA use was reported by 95% (40/42) of the asthma group and most (60.5%, 25/42) were on at least one controller medication. The most common medication was inhaled corticosteroids (ICS), which was reported by 38% (16/42) of the asthma group, followed by ICS and long-term beta-agonist combination (29%, 12/42), and leukotriene modifier (21%, 9/42). No significant difference in control medication use was found between the symptomatic and nonsymptomatic groups. Only 2 participants (5%, 2/42) reported oral steroids as a current medication.

### Coughs in the Asthma Group Monitored by the Device

Descriptive analysis was conducted on data from the asthma group excluding 3 asthma teens for which no data were recorded in the device due to unknown mechanical issues. All 39 teens with asthma used the device for a mean 8.26 (SD 1.47) consecutive days (median 8, range 5-14 days) and each teen used the device for a mean 19.4 hours/day (SD 1.71; median 19.7, range 15-22 hours). When the number of coughs was compared for morning (6 am-11:59 am), afternoon/evening (noon-10 pm), and bedtime (10 pm-6 am), a greater number of coughs were registered during afternoon/evening compared to morning and bedtime (*P*=.01 and *P*=.004, respectively). The number of coughs was not significantly different by gender or age.

### Concurrent Validity

#### Cough Counts and Fractional Exhaled Nitric Oxide and Pulmonary Function

No significant correlations were found between the device’s cough data and FeNO. The number of coughs was negatively associated with FEV_1_ (*r*=-.26, *P*=.05) and FVC (*r*=-.31, *P*=.02) at enrollment indicating that the higher number of coughs was associated with poor pulmonary function.

#### Cough Counts and Daily Symptom Diary Data and Visual Analog Scale

Cough counts showed a positive association with limited activities (*r*=.46, *P*=.01) and shortness of breath (r=.29, *P*=.07). Cough counts showed significant associations with symptoms as measured by VAS at nighttime (*r*=.40, *P*=.02) and daytime (*r*=.38, *P*=.02).

#### Cough Counts and Asthma Control and Quality of Life

Table 1 shows correlations between cough counts and asthma control and quality of life during the trial. Cough counts were negatively associated with asthma control and overall quality of life, and the activity and symptom subscales of quality of life.

**Table 1:** Associations between the number of coughs and asthma control and quality of life.

Associated variables		*r*	*P*
Asthma control		-.41	.01
**Quality of life total**		-.28	.08
	Activity subscale	-.27	.09
	Symptoms subscale	-.29	.07
	Emotional function subscale	-.26	.11

#### Cough Counts and Health Care Utilization

There was no association between cough counts and health care use in the 3 months before the trial. However, cough counts showed positive association with health care use during the 7-day trial (*r*=.61, *P*<.001) indicating that higher cough counts were associated with the higher use of health care services, particularly with the number of days of hospitalization (*r*=.72, *P*<.001) and office visits (*r*=.72, *P*<.001). Cough counts were also positively associated with the number of missed school days (*r*=.70, *P*<.001).

### Predictive Validity

Greater number of coughs was correlated with poor asthma control and quality of life and higher use of health care services 3 months later (Table 2).

**Table 2:** Correlations between the number of coughs and asthma control, quality of life, and health care utilization at 3 months after the 7-day trial.

Dependent variables		*r*	*P*
Asthma control		-.49	.002
**Quality of life total**		-.47	.004
	Activity subscale	-.45	.006
	Symptoms subscale	-.45	.006
	Emotional function subscale	-.44	.007
Health care utilization		.55	.02

Table 3 presents the extent to which cough counts predicted asthma control, quality of life, and health care utilization after adjusting for age, gender, race, and family income. Coughs significantly predicted asthma control, and the regression model explained 42% of the variance in asthma control. Cough counts also significantly predicted the quality of life total score (β=-.55, *P*=.001) and each of the subscales including activity, symptoms, and emotional function 3 months later. Explained variance in quality of life was 38% for the quality of life total score, and 28%, 35%, and 41% for activity, symptoms, and emotional subscales, respectively. Health care utilization during the 3 months following the 7-day trial was significantly predicted by cough counts (β=.74, *P*=.004), explaining 76% of the variance. Particularly, coughs were associated with ED visits (*r*=.47. *P*=.004), asthma specialist visits (*r*=.45, *P*=.005), and office visits due to worsening asthma (*r*=.39, *P*=.02) that occurred in the 3-month period after the trial.

**Table 3:** Asthma control, quality of life, and health care utilization predicted by coughs and demographic variables.

Predictors	Asthma control		Quality of life								Health care utilization	
	β	*P*	Total		Activity		Symptoms		Emotional function		β	*P*
			β	*P*	β	*P*	β	*P*	β	*P*		
Cough counts	-.48	.003	-.55	.001	-.50	.005	-.50	.003	-.58	<.001	.74	.004
Age in years	.05	.73	.11	.47	.11	.53	.07	.65	.16	.29	.19	.42
Gender (1=female)	.08	.64	-.19	.28	-.19	.30	-.22	.20	-.11	.51	-.26	.25
Race (1=nonwhite)	.36	.14	-.03	.91	.10	.71	.04	.88	-.20	.43	.03	.90
Household income	.06	.83	.03	.92	.03	.93	.11	.68	-.09	.72	-.43	.17

### Sensitivity and Specificity

**Figure 1:**
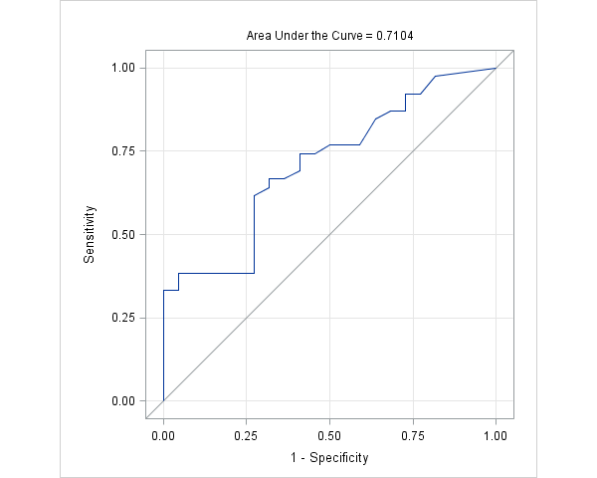
Receiver operating characteristic (ROC) curve analysis for predictive values of coughs.

The capacity of the device to distinguish participants with asthma from those without asthma was assessed using ROC curve analysis. The ROC curve for the presence of asthma diagnosis had an AUC of 0.71 (95% CI 0.58-0.84). The AUC was significantly different from chance (χ^2^_1_=9.7, *P*=.002). The optimal cutoff value of the device was 0.56, with 51.3% sensitivity and 72.7% specificity (Figure 1). The cutoff value discriminating teens with asthma from those without asthma was translated into 0.83 coughs/hour or 19.92 coughs/day.

### Overview of the Study Findings

Table 4 summarizes findings pertaining to the validity of the device. Most of the expected relationships between ADAM data and conventional measures of asthma were substantiated by our findings.

**Table 4:** Overview of study findings and expected relationships between cough counts and measures of asthma.

Types of validity		Expected relationships	Statistical method	Findings
**Concurrent validity**				
	Cough counts and FeNO and lung function	Positive association with FeNO; negative association with lung function	Correlation	No significant correlations with FeNO; cough counts were negatively associated with FEV_1_ and FVC
	Cough counts and symptom diary data and VAS	Positive association	Correlation	Associated with limited activities and approached significance for shortness of breath and number of rescue medications use in the past 24 hours
	Cough counts and asthma control	Negative association	Correlation	Cough counts were negatively associated with asthma control
	Cough counts and quality of life	Negative association	Correlation	Approached significance with quality of life, activity, and symptom subscales
	Cough counts and health care utilization	Positive association	Correlation	No association between cough counts and health care use before the 7-day trial; however, cough counts showed positive association with health care use during the 7-day trial
**Predictive validity**				
	Cough counts and asthma control and quality of life 3 months later	Cough counts predicting asthma control and quality of life	Multiple regression	Coughs predicted asthma control 3 months later explaining 42% of the variance in asthma control. Coughs predicted the quality of life total score and each of subscales 3 months later, explaining variance in quality of life, which ranged from 28% to 41%
	Cough counts and health care utilization 3 months later	Cough counts predicting health care utilization	Multiple regression	Coughs predicted health care utilization 3 months later explaining 76% of the variance in health care utilization
**Clinical prediction**				
	Area under the curve		ROC curve analysis	0.71 (95% CI 0.58-0.84)
	Cutoff point		ROC curve analysis	0.56 (0.83 coughs/hour or 19.92 coughs/day)
	Sensitivity	Discrimination of positive asthma diagnosis by a cutoff	ROC curve analysis	51.3% sensitivity
	Specificity	Discrimination of negative asthma diagnosis by a cutoff	ROC curve analysis	72.7% specificity

## Discussion

This study examined the validity of ADAM, an investigational device that automatically monitors coughs continuously in adolescents with asthma. To our knowledge, ADAM is the first fully automated portable device facilitating continuous monitoring of the frequency of coughs that involves real-time processing, analysis, recording, and displaying of symptoms. Previously, we reported technical details [35] and the acceptance of the device by teen users [34]. In these earlier reports, we demonstrated the feasibility of developing an algorithm for coughs, but not for wheezes due to the wide intrapersonal and interpersonal variability of the acoustic signature of wheezing. Therefore, ADAM was evaluated solely as a cough-monitoring device at this time. Coughs are widely recognized as a key symptom of asthma [9,44] and the most common symptom of uncontrolled asthma in children and adolescents [7,45-47]. Asthma patients report coughs as the most troublesome symptom in their lives and as a symptom of greater importance [48]. Given the importance of coughing in asthma, ADAM can be a clinically useful monitoring tool not only for the symptom itself, but also for symptom burdens on individuals. In this paper, we examined the validity of the device as an asthma-monitoring tool and its capacity to discriminate asthma cases from controls.

### Principal Results

Overall, we found positive temporal correlations, albeit low to moderate, between device data (ie, cough counts) and conventional measures of asthma symptoms and symptom control. Similarly, previous studies reported modest correlations between objective cough rates and subjective measures specific to cough (VAS for cough and cough scores) in individuals with asthma [49,50]. In our study, the generic self-report measures of asthma symptoms made it difficult to assess the degree of agreement between the device’s cough counts and the amount of coughs perceived by individuals. Nonetheless, when conceptualizing coughs as an indicator of asthma condition, use of generic measures of asthma control to establish the device’s validity as an asthma-monitoring tool can be justified. The demonstrated relationships between cough counts and concomitantly assessed activity limitation and other symptoms as well as overall asthma control provide support for the validity of the device as a tool for overall asthma monitoring rather than simply for cough. As in another study [49], the relationship between cough counts and quality of life suggest that experiencing coughing can take a toll on quality of life in teens with asthma. Moreover, the positive relationships between cough counts and health care utilization and school absenteeism during the trial could be further evidence that cough counts as measured by ADAM is a compelling indicator of asthma morbidity.

Unlike earlier reports of no relationship between spirometry data and coughs measured by an objective method [49,51] or self-report [52], we found that the cough counts by ADAM were associated with poor pulmonary function (as indicated by FEV_1_ and FVC), suggesting that the number of coughs are indicative of airway obstruction. However, consistent with an earlier study [49], we were unable to establish the relationship between coughing and airway inflammation measured by FeNO. This may be because most (>50%) of the asthma group were asymptomatic (ie, no indication of active airway inflammation) before and during our trial period. Replication of the trial using a large number of patients with active symptoms is needed to determine the nature of relationship between coughs and airway inflammation.

This study demonstrated strong evidence for predictive validity of the device. After adjusting for sociodemographic factors, cough counts were predictive of asthma morbidity and quality of life at 3 months after the 7-day trial. The cough count predicted poor asthma control and quality of life at 3-month post-trial, accounting for 42% and 38% of the variance, respectively. Health care services used during 3 months after the trial were predicted by the cough count by our device and explained as much as 76% of the variance in acute health care utilization 3 months later. In an earlier study [53], worsening coughing was found to be predictive of severe asthma after 9 years in adult patients. Our findings support not only ADAM’s predictive validity, but also coughs as an important harbinger for upcoming deterioration of teens’ asthma morbidity that could undermine their overall well-being and impose serious burdens on the health care system. As such, the findings underscore the importance and need for a monitoring device such as ADAM that can increase patients’ awareness of the patterns of cough for early detection and has the potential for preventing serious and costly future consequences of asthma morbidity.

The device’s capacity to discriminate correctly the asthma group from those without asthma was assessed to determine sensitivity and specificity. We found poor diagnostic sensitivity of ADAM; that is, the chances of correctly identifying those with asthma were only 51% using 20 coughs/day as a cutoff. The poor diagnostic sensitivity of the device may have been due to the asymptomatic state of more than 50% of the asthma group in which the average cough counts were below the cutoff making it difficult for the device to appropriately identify asthma cases. In the future, studies maximizing differences between subsamples in symptom presentation will be essential for adequately assessing the sensitivity of the device. By contrast, ADAM demonstrated relatively better specificity such that the device correctly classified those who did not have asthma for 73% of cases. Using the device in an environment replete with everyday noises may result in a high number of cough counts exceeding the cutoff, even in those without asthma; thus, incorrectly excluding them from the nonasthma group. We observed occasional false events (2 coughs/hour) depending on environmental noises (70% sensitivity of the cough algorithm) [35], which is strikingly similar to 2.5 events/hour by the Leicester Cough Monitor [54]. Given the poor capacity to correctly classify patients with asthma, ADAM is *not* suitable for determining a clinical diagnosis of asthma, but is intended solely for monitoring cough in those with a confirmed asthma diagnosis. Like any monitoring device, false alarms are still an issue because these can cause unnecessary concerns or undermine users’ confidence in the monitoring tool. Therefore, further optimizing the accuracy of the monitoring device is warranted by refining the algorithm and adopting noninvasive techniques to minimize the influence of environmental noises (eg, direct application of an adhesive microphone on the chest wall).

### Study Limitations

Several limitations to this study’s design warrant caution. Because the device stored only the number of coughs without sound recording, validating the accuracy of cough counts through the manual confirmation of corresponding cough events was not done. Except for pulmonary function tests and FeNO, we primarily used self-report measures that were not specific to coughs to establish the validity of the device. The generic nature of the measure may have contributed to some of the nonsignificant correlations between the measures and device data. Although self-report measures are inherently subject to recollection bias, we attempted to address the challenge by strategically collecting daily symptom data electronically. In health care utilization data, we observed more than 90% agreement between self-report and medical record review. Therefore, it appears that recollection bias played little influence on our validity outcomes. Nonetheless, future research is needed to evaluate the validity of the device by simultaneously recording raw cough sounds and by using cough specific measures.

Moreover, distinction between the asthma and nonasthma groups was blurred because most of the asthma group did not present active symptoms during the trial, which may account for the device’s poor sensitivity. Also, use of the small and convenient sample of adolescents limited generalizability of the findings. In addition, the length of the 7-day trial might not be long enough to observe any meaningful changes in asthma symptoms, health care utilization, or biological measures including FEV_1_ and FeNO. A longer observation period is warranted to assess the extent to which the device adequately captures changes over time in symptom patterns and other measures of asthma status. Lastly, there were a few technical challenges and limitations to the optimal operation of the device, which we discussed in our earlier report [34].

### Comparison With Prior Work

Several approaches have been attempted to develop technologies to monitor symptoms objectively, particularly coughs [45,49,50,54-58]. However, these existing approaches are considered unsuitable for ambulatory monitoring of symptoms due to practical challenges, including the laborious and time-intensive nature of processing audio data (so not fully automated and unable to provide real-time feedback to users), inability to monitor continuously beyond 24 hours, and the conspicuous appearance of the systems. For instance, the Leicester Cough Monitor [54,59,60] uses a similar sound recognition technical approach to that of ADAM. Unlike ADAM, in the Leicester Cough Monitor, the ambulatory component of the system consists strictly of an audio recorder and audio analysis is performed offline, which can take approximately 1 hour to process a 24-hour audio recording. This differs fundamentally from ADAM in which all of the processing/annotation of audio data are performed instantly on the mobile device and the results (cough counts) are provided as feedback in real time. Other cough detection systems [61] are not intended for ambulatory use or have yet to be validated in vivo for an extended observation period. In that sense, ADAM is the first ambulatory cough-monitoring device on a consumer mobile system with a capacity to fully automatize continuous real-time processing. This is also the first attempt to evaluate the validity of a cough detection device as a symptom-monitoring tool for several consecutive days in adolescents with asthma. Using a popular mobile device, such as iPod, as a platform for ADAM was well received by adolescents with asthma [34] and the majority of participants used the device daily for a week or longer period supporting ADAM as a sustainable asthma-monitoring tool for adolescents.

### Conclusions

Overall, this study demonstrated the validity of ADAM as a symptom-monitoring device in teens with a confirmed asthma diagnosis. Poorly controlled asthma takes a toll on teens’ overall health and quality of life as well as on the health care system due to an increased economic burden associated with the use of urgent types of health care services. ADAM can potentially mitigate the adverse consequences by helping users detect and treat early symptoms before advancing to a poorly controlled state. The device is useful in increasing understanding about one’s current status of asthma control and in predicting asthma morbidity and quality of life for the near future. Such information can make the users become aware of symptoms and triggers and enable them to take appropriate and timely actions to address symptoms or prevent further deterioration of their symptoms. Objective symptom information from the device would be clinically useful in establishing optimum treatment plans and evaluating treatment effects. ADAM can be particularly useful and effective in monitoring symptoms occurring at night when environmental noises are minimal. Nighttime coughs were more common than wheezing [62] and worsening of asthma symptoms often occurs during nighttime [63]. As such, nighttime symptoms are often indicative of poorly controlled asthma [31,64], but patients often do not recognize or tend to neglect to report nighttime symptoms [65-68]. ADAM can be an invaluable tool that monitors nighttime symptoms, which would provide important clinical insight into the degree of symptom control and the response to asthma treatment.

Nonetheless, we caution that the device should not be considered as a diagnostic tool and its application to a broader age range remains to be evaluated. Although we suggest coughing as an important indicator of asthma morbidity, understanding the symptom in the context of other information, such as activity level and medication use, can potentially augment its clinical value and relevance. Originally, ADAM was designed to monitor symptoms and activity levels simultaneously capitalizing on the host system’s (iPod) built-in accelerometer to offer an insight into the nature of the relationships between symptoms and activity levels (eg, exercise-induced asthma). However, continuously recording and processing sounds and activity simultaneously quickly drained the platform battery, posing challenges for long-term uninterrupted sound processing. More study is needed to reassess the feasibility of concurrent operation of these 2 applications with optimized code and application power management. ADAM also allowed users to record the use of medications throughout the day to help them systemically review changes in symptom patterns in relation to medication use. This can motivate users’ adherence to treatment plans (when they noted symptom reduction after medication) or inform users or providers of the need for adjusting current medication or dosage (when no relief from symptom was achieved after medication). A clinical trial is needed to examine the effects of the device’s medication tracking function on users’ medication adherence.

ADAM represents an important undertaking in the field of mobile health (mHealth) that has exploded in the past decade to improve health outcomes and patients’ self-management capacity [69-71]. The application of mobile technologies in daily assessment of asthma symptoms can be a particularly attractive option for adolescents. Yet to be determined is the extent to which the appeal of mobile technology can translate into better symptom monitoring, awareness, and self-management behaviors, which ultimately leads to improved asthma outcomes.

### Potential Clinical Application of ADAM: Sample Case Scenario

The following sample clinical scenario demonstrates the potential application of ADAM in clinical practice:

JD is a 16-year-old boy with moderate persistent asthma and a history of several emergency visits for asthma each year. His provider had prescribed a daily controller medication for him, but he rarely uses it because he has not perceived any significant benefit. He reports not sleeping well, which causes him to feel poorly rested in the morning. He relies heavily on albuterol with several self-administered doses each day. After a discussion with his provider, JD agreed to use ADAM for a month. At a follow-up visit, the provider and JD reviewed the symptom pattern displayed on the screen of the mobile phone monitor. Data from the device revealed significant coughs, predominantly at night, which JD was unaware. After discussing the results, JD agreed to use the controller medication daily to see if the symptoms improved. After 1 month, JD repeated his symptom monitoring with ADAM and discovered his coughing events were significantly decreased compared to the prior monitoring period. In addition, JD was alerted by ADAM when his symptoms became out of control, which allowed him to take precautions and closely follow his asthma action plan. He agreed to continue to use the controller medication for another 2 months and to use ADAM intermittently to help track his symptoms.

## Abbreviations

ADAM: Automated Device for Asthma Monitoring
ATS: American Thoracic Society
AUC: area under the curve
FeNO: fractional exhaled nitric oxide
FEV_1_: forced expiratory volume in first second of expiration
FVC: forced vital capacity
ICS: inhaled corticosteroids
NHLBI: National Heart, Lung, and Blood Institute
PAQLQ: Pediatric Asthma Quality of Life Questionnaire
ROC: receiver operating characteristic
SABA: short-acting beta agonists
VAS: Visual Analog Scale
